# Single-Cell Sequencing Reveals the Crosstalk Between MuSCs and FAPs in Ruminant Skeletal Muscle Development

**DOI:** 10.3390/cells15020206

**Published:** 2026-01-22

**Authors:** Yuan Chen, Yiming Gong, Xiaoli Xu, Meijun Song, Xueliang Sun, Jing Luo, Jiazhong Guo, Li Li, Hongping Zhang

**Affiliations:** Key Laboratory of Livestock and Poultry Multi-omics, Ministry of Agriculture and Rural Affairs, College of Animal Science and Technology, Sichuan Agricultural University, Chengdu 611130, China; s20163653@stu.sicau.edu.cn (Y.C.); 2024102012@stu.sicau.edu.cn (Y.G.); 2022102009@stu.sicau.edu.cn (X.X.); 2023102002@stu.sicau.edu.cn (M.S.); 2023102018@stu.sicau.edu.cn (X.S.); luojing@stu.sicau.edu.cn (J.L.); jiazhong.guo@sicau.edu.cn (J.G.)

**Keywords:** single-cell RNA sequencing, skeletal muscle, MuSCs, FAPs, intercellular interaction, ruminants

## Abstract

Skeletal muscle orchestrates a remarkable journey from embryonic formation to age-related decline, yet its cellular intricacies in goats remain largely uncharted. We present the first single-cell RNA sequencing (scRNA-seq) atlas of the longissimus dorsi muscle from goats, profiling 120,944 cells across 14 developmental stages from embryonic day 30 (E30) to 11 years postnatal (Y11). We focused on skeletal muscle satellite cells (MuSCs) and fibro-adipogenic progenitors (FAPs), identifying a unique MuSCs_*ACT1*_high subpopulation in early embryogenesis and a senescence-associated MuSCs_*CDKN1A*_high subpopulation in later developmental stages. In FAPs, we characterized the early-stage FAPs_*MDFI*_high subpopulation with differentiation potential, which further exhibited the capacity to commit to both adipogenic and fibrogenic lineages. Transcription factor analysis revealed strikingly similar regulatory profiles between MuSCs and FAPs, suggesting that these two cell types are governed by shared signaling pathways during development. Cell–cell interaction analysis demonstrated that the DLK1-NOTCH3 ligand-receptor pair plays a critical role in enabling early embryonic FAPs to maintain the quiescent state of MuSCs. This dynamic single-cell transcriptomic atlas, spanning 14 developmental stages of skeletal muscle in ruminants for the first time, provides a valuable theoretical foundation for further elucidating the differentiation of skeletal muscle satellite cells and fibro-adipogenic progenitors in ruminants.

## 1. Introduction

Skeletal muscle is a highly specialized tissue composed of multinucleated myofibers that originate from mononuclear myoblasts during embryonic development [1]. Myoblast fusion is essential not only for prenatal myogenesis but also for adult skeletal muscle regeneration following injury [1]. During the embryonic and fetal development stages (collectively referred to as prenatal), abundant muscle progenitor cells (MPCs) proliferate rapidly and fuse to form myofibers, supporting rapid muscle growth. Postnatally, MPC activity declines, and these cells enter a reversible quiescent state beneath the basal lamina of myofibers, becoming muscle satellite cells (MuSCs). Under the combined influence of external mechanical stimulation and endogenous signals from FAPs, quiescent skeletal muscle satellite cells switch from a dormant state to an activated state [2,3]. This activation is accompanied by profound global transcriptional reprogramming, including upregulation of myogenic regulators such as *Myf5* and *MyoD* [4,5].

MuSC fate is governed by intricate internal (e.g., cell–cell interactions) and external (e.g., niche signals) mechanisms, resulting in substantial heterogeneity within the MuSC population and across skeletal muscle cell types [6]. Traditional bulk RNA sequencing provides averaged tissue-level insights, whereas single-cell RNA sequencing (scRNA-seq) has revolutionized our ability to resolve distinct cell states, subpopulations, and precise gene expression profiles in complex tissues [7,8]. scRNA-seq has been instrumental in dissecting cellular heterogeneity in skeletal muscle, revealing major populations including MuSCs, fibro-adipogenic progenitors (FAPs), endothelial cells, tenocytes, smooth muscle cells, immune cells (e.g., macrophages, B and T cells), and neural cells [5,9,10]. In 2017, Porpiglia et al. recognized surface markers (*CD104*) that uniquely distinguish myogenic stem cells from progenitor cells in skeletal muscle in vivo and delineated myogenic trajectories during recovery from acute muscle injury [11]. Sequencing of mouse skeletal muscle during muscle repair and stabilized at a moderate level postnatally identified two types of MuSCs that can be distinguished into resting and activated states, with greatly differing *Pax7* and *MyoD* levels [5].

Beyond MuSCs, non-myogenic cells play critical supportive roles in muscle repair and regeneration. FAPs, marked by *PDGFRα*, *Sca1*, and *CD34*, secrete pro-regenerative factors (e.g., fibronectin, IGF-1, matrix proteins, and growth factors) that modulate myogenic cell fate, clear debris, and maintain structural integrity during regeneration [11,12,13]. Recent studies have further highlighted dynamic FAP subpopulations across regeneration timelines, underscoring their regulatory mechanisms in response to injury [14]. In the same way, MuSCs can determine the fate of other cells in the tissue. For example, it can affect the gene expression of fibroblasts and the process of differentiation [15]. Recent studies have shown that FAPs provide a supportive microenvironment for MuSCs during skeletal muscle development and aging. FAPs promote myogenesis in MuSCs by releasing miR-127-3p via extracellular vesicles (EVs) [16]. Conversely, MuSCs release miR-206-3p and miR-27a/b-3p through EVs to inhibit adipogenic differentiation of FAPs [16]. The bidirectional crosstalk between FAPs and MuSCs exerted significant influence on adipogenesis, intramuscular adipose infiltration, and the process of skeletal muscle regeneration [17]. Ma et al. [18] found that FAPs significantly influence the regeneration of MuSCs through the FGF7–FGFR2 ligand–receptor pair. Exogenous administration of FGF7 promoted muscle regeneration following cardiotoxin (CTX)-induced injury and ameliorated age-related myopathy induced by D-galactose (D-gal).

Despite these advances in rodent and human models, significant knowledge gaps remain in ruminant skeletal muscle biology, particularly in economically important species such as goats. Comprehensive single-cell atlases capturing long-term developmental trajectories (embryonic to aged stages) were largely absent for goats, limiting understanding of species-specific MuSC and FAP heterogeneity, regulatory convergence, and crosstalk dynamics. Moreover, it remains largely unknown whether and to what extent MuSCs exhibit phenotypic heterogeneity at the single-cell level within a single tissue and how this heterogeneity manifests across different developmental ages or in response to aging.

In this report, we generated the first single-cell RNA sequencing atlas of the longissimus dorsi muscle in goats, profiling 120,944 cells across 14 developmental stages from embryonic day 30 (E30) to 11 years postnatal (Y11). Focusing on MuSCs and FAPs, we characterized subpopulation composition, pseudotime developmental trajectories, and notable transcriptional similarities between these two cell types. We identified a unique early embryonic MuSCs_*ACT1*_high subpopulation and a senescence-associated MuSCs_*CDKN1A*_high subpopulation in later stages. In FAPs, we delineated an early-stage FAPs_*MDFI*_high subpopulation with bipotent adipogenic and fibrogenic potential. Transcription factor network analysis revealed strikingly shared regulatory landscapes between MuSCs and FAPs, while cell–cell interaction analysis uncovered the pivotal role of the DLK1-NOTCH3 ligand–receptor pair in maintaining MuSC quiescence during early embryogenesis. This comprehensive developmental atlas provides a foundational resource for understanding MuSC and FAP dynamics in ruminants, offering insights into muscle growth, meat quality, and regenerative mechanisms in livestock.

## 2. Materials and Methods

### 2.1. Animal Ethics and Sample Collection

All animal experiments in this research were conducted in strict accordance with the Regulations for the Administration of Affairs Concerning Experimental Animals (Ministry of Science and Technology, Beijing, China) and were approved by the Animal Care and Use Committee of Sichuan Agricultural University (approval number: 20200536).

The Chengdu gray goats used in this study were obtained from the Chengdu Gray Goat Breeding Farm (Chengdu, China). Embryos were collected from pregnant females at designated gestational stages following artificial insemination and cesarean section; fetuses were humanely collected at 30, 45, 60, 75, 90, 105, 120, and 135 days of gestation (E30, E45, E60, E75, E90, E105, E120, E135) by cesarean section. Postnatal animals were fasted for 24 h prior to humane slaughter by jugular vein exsanguination (B0, B28, B90, Y1, Y3, and Y11).

Longissimus dorsi (LD) muscle samples for single-cell sequencing were collected using an 8-point mixed sampling method: one sample each from the left and right sides at the fourth and tenth thoracic vertebrae and the second and fifth lumbar vertebrae. For the early embryonic stages (E30, E45, and E60), due to the small size of the fetuses, all available LD muscle tissue along the dorsal midline was collected bilaterally to compensate for the inability to perform the standard 8-point sampling. The collected tissue was then thoroughly pooled and mixed. All harvested LD muscle samples were immediately rinsed with pre-chilled PBS containing triple antibiotics (4% penicillin-streptomycin solution and 4% gentamicin solution) at 4 °C, placed in tissue preservation solution, and transported on ice for single-cell library preparation. For histological sectioning, LD muscle tissue near the thoracolumbar region was excised as intact strips approximately 1 cm in length, fixed in 4% paraformaldehyde (Sorlabio, Beijing, China), and processed for sectioning and analysis. Remaining samples were snap-frozen on dry ice and stored at −80 °C as backups.

### 2.2. Histological Processing and H&E Staining

LD muscle tissues were fixed in 4% paraformaldehyde (Sorlabio, Beijing, China) at 4 °C overnight. The following morning, tissues were dehydrated in an ethanol series and incubated in xylene for 30 min. Samples were embedded in paraffin blocks and sectioned at 5–7 μm using a Leica RM2255 microtome (Leica, Nussloch, Germany). Sections were transferred to APES-treated slides (ZSGB-BIO, Beijing, China) to prevent detachment. For hematoxylin and eosin (H&E) staining, slides were deparaffinized in 100% xylene for 30 min, rehydrated in an ethanol series, stained with hematoxylin for 7 min, and washed twice with distilled water for 5 min. Slides were then rinsed with 1% HCl (*v*/*v*) ethanol for 3–5 s, washed with 45 °C water for 5 min, stained with 1% eosin ethanol, and rinsed with absolute ethanol for 10 min. Finally, slides were mounted with neutral resin and imaged under an optical microscope (McAudi Industrial Group Co., Ltd., Sichuan, China). The diameter distribution of LD muscle fibers across 14 developmental stages was analyzed, with 200 measurements examined in 10 fields of view using ImageJ software (v1.8.0).

### 2.3. Single-Cell RNA-Seq Preparation and Library Construction

Fresh LD muscle tissues were rinsed with PBS, finely minced, and enzymatically digested in 0.125% trypsin/EDTA solution at 37 °C for 30 min. After centrifugation, the precipitate was digested with 2 mg/mL collagenase I/II (Sigma, St Louis, MO, USA) for 1 h. Cell suspensions were filtered through a 70 μm nylon cell strainer (BD Falcon, BD Biosciences, San Jose, CA, USA) to remove debris. After centrifugation at 300 rpm for 3 min, the precipitate was resuspended in PBS, and cell suspensions with viability > 80% were used. Dead cells were removed using the Dead Cell Removal Kit (Miltenyi Biotec GmbH, Cologne, Germany) to enhance sorting efficiency. Cell number and viability were verified using a Countess II Automated Cell Counter (Thermo Fisher Technology (China) Co., LTD, Shanghai, China). Single-cell suspensions were used for library construction following the 10 ×Genomics single-cell RNA sequencing platform (10× Genomics, Pleasanton, CA, USA). Suspensions were diluted to a final concentration in DMEM with 10% FBS or DMEM/F12 and loaded onto a Chromium Controller to generate single-cell gel bead emulsions, targeting 6000–10,000 cells depending on the tissue and embryonic stage. Eight libraries were generated for embryonic stages and six for postnatal stages. Single-cell 3′ RNA-seq libraries were prepared using the Chromium Single Cell 3′ Reagent v2 Chemistry Kit (10× Genomics (Shanghai) Co., Ltd., Shanghai, China) according to the manufacturer’s instructions.

### 2.4. Sequencing Quality Control, Filtering, and Integration

Sequencing was performed on an Illumina Novaseq 6000 system, generating paired-end 150 bp (PE150) reads. Quality control of the raw data, base quality distribution, and content was performed using FastQC. Raw base call (BCL) files were converted to FASTQ files using the “cellranger mkfastq” function from Cell Ranger Single Cell Software Suite (v4.0, 10× Genomics (Shanghai) Co., Ltd., Shanghai, China). FASTQ files were indexed with the “mkref” function using the Capra hircus v1.0 reference genome (NCBI: GCF_001704415.1). Gene expression and feature barcoding reads were counted using the “cellranger count” function. Output files containing gene expression matrices and barcode information were used for downstream analysis. Over 90% of reads aligned to the goat genome, with Q30 base rates exceeding 90%. The mean number of reads per cell ranged from 44,002 to 89,570, and 14,401 to 17,002 genes were detected per sample. The estimated number of cells ranged from 6270 to 11,688 across 14 samples (Supplemental Table S1).

Filtered gene–barcode matrices from Cell Ranger were analyzed using Seurat (v5.0) in R (v4.3.2). Quality control removed low-quality cells based on UMI counts, gene numbers, and mitochondrial/ribosomal gene percentages, with the following criteria: 500 < nCount_RNA < 30,000, nFeature_RNA > 200, percent.rb < 30, and percent.mt < 25. Doublets were filtered using DoubletFinder (v2.0.3). After quality control, 114,280 cells remained, with capture rates ranging from 5720 to 10,566 cells per sample (Supplemental Table S2). Gene expression data were log-transformed and normalized using Seurat’s “NormalizeData” function with a scale factor of 10,000.

### 2.5. Single-Cell RNA-Seq Data Processing and Analysis

All analyses were performed in R v4.3.2. Raw FASTQ files were aligned to the Capra hircus v1.0 reference genome (NCBI: GCF_001704415.1) using Cell Ranger v4.0 (10× Genomics). Data from 14 developmental stages were integrated in Seurat v5.0; the top 3000 highly variable genes were selected via FindVariableFeatures (“vst” method), scaled with ScaleData, and batch-corrected using CCA via FindIntegrationAnchors and IntegrateData. Dimensionality reduction used the first 100 PCs for PCA and UMAP. Graph-based clustering was performed with FindNeighbors and FindClusters (resolution = 0.6). Cluster markers were identified using FindAllMarkers (Wilcoxon test; log_2_FC > 0.25, min.pct = 0.1, adj. *p* < 0.05) and annotated using canonical markers, PanglaoDB, and functional enrichment of the top 100 genes, confirmed by ClusterTree.

Differentially expressed genes were detected with FindMarkers (Wilcoxon test; same thresholds). GO (BP/MF/CC) and KEGG pathway enrichment were conducted using clusterProfiler v4.6.2 with org.Cg.eg.db/org.Hs.eg.db annotations; *p*-values were adjusted via Benjamini–Hochberg procedure (adj. *p* < 0.05). Results were visualized with enrichplot and ggplot2.

Subclustering of MuSCs, FAPs, and ENDs was performed using SubsetData and reprocessed at resolution = 0.1; subsets were named by the highest-expressed gene among the top 10 HVGs. The pseudo-temporal analysis using Monocle3 enables a deeper understanding of the precise differentiation trajectories of cells from a developmental perspective. Gene regulatory networks were inferred using SCENIC: TF–target links were ranked by GENIE3 (top 5 TFs/gene), modules built, and motifs enriched via cisTarget. Cell–cell communication was analyzed with CellChat v2.1.2 across all stages, reintegrating data into four developmental groups via Pearson clustering and PCA of cell-type proportions (13 cell types, complete-linkage). Networks were annotated using CellChatDB.human.

## 3. Results

### 3.1. Histological Changes in Skeletal Muscle

**Figure 1:**
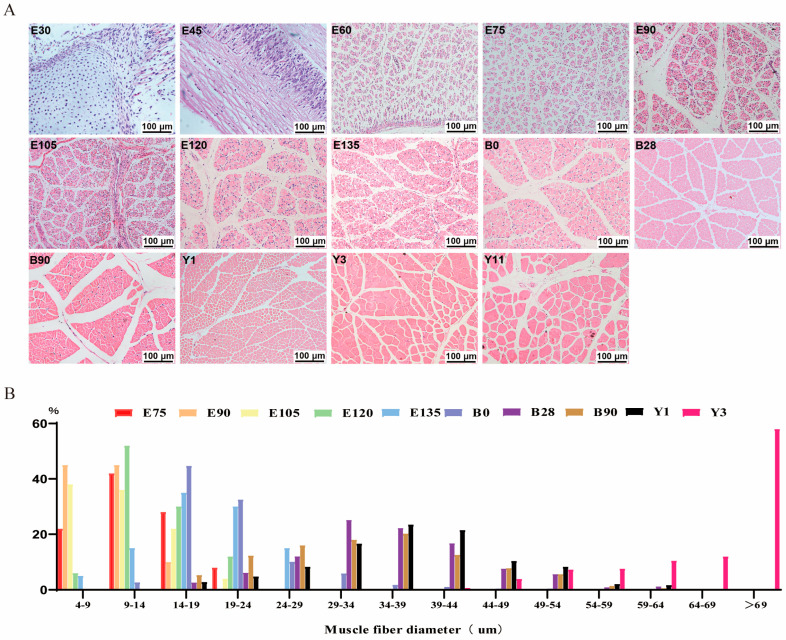
The specificity of skeletal muscle tissue of the goat in 14 periods. (**A**). HE staining of LD muscle sections at different periods. Scale bars: 100 μm. (**B**). Muscle fiber diameter distribution of skeletal muscle sections at 10 different periods from E75 to Y3.

To systematically investigate morphological changes in the skeletal muscle of goats, samples from 14 developmental stages, spanning embryonic (E30, E45, E60, E75, E90, E105, E120, E135) and postnatal periods (B0, B28, B90, Y1, Y3, Y11), were analyzed (Supplementary Figure S1A). Chronologically ordered HE-stained sections revealed dense connective tissue with sparse myocytes and no mature fibers at E30–E60. Distinct myocytes first emerged at E75, fusing into hollow tubular primary myotubes. By E90, mature muscle fibers formed fully, characterized by increased secondary myofibers and residual primary myotubes; thereafter, progressive hypertrophy of muscle fiber diameter was observed across postnatal stages (B0 to Y11) (Figure 1A).

This study statistically analyzed changes in skeletal muscle fiber diameter in goats, revealing developmental trends (Figure 1B). From the embryonic stage onward, the median diameter increased progressively with age. During the mid-to-late embryonic period (E75–E120), fiber diameters showed insignificant variation and were predominantly below 25 μm. From late embryogenesis to postnatal stages, diameter variability gradually intensified, with B28-Y1 stages exhibiting similar distribution patterns (concentrated at 24–49 μm). The Y3 stage displayed a significantly larger median diameter than all other stages. These findings demonstrate that myocyte hypertrophy primarily occurs during late embryogenesis and postnatal development. Earlier research on Jianyang Big-Ear goats similarly reported significant growth rate differences across stages E45–E60, E60–E105, and E105–B321 [19]. Collectively, histological examination confirmed substantial transformations in the skeletal muscle of goats.

### 3.2. Cellular Heterogeneity of Skeletal Muscle Across Developmental Stages

**Figure 2:**
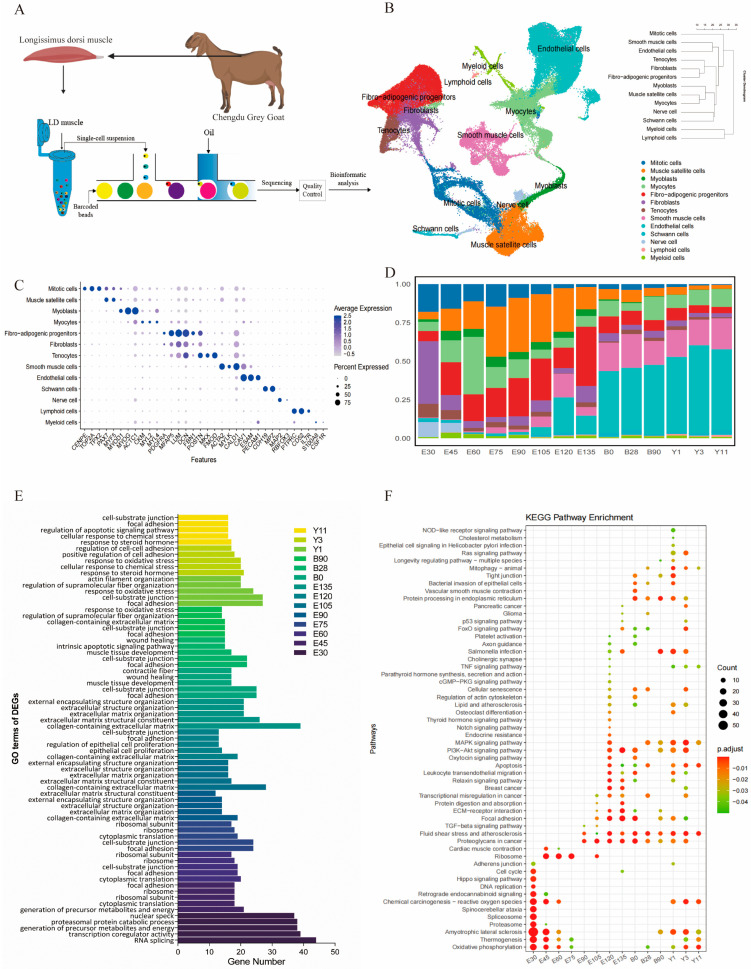
Single-cell atlas and differential gene analysis of goat skeletal muscle in 14 periods. (**A**). Schematic workflow of scRNA-seq for goat LD muscle. (**B**). UMAP of both 14 periods and samples after calibration of cell cycle effects. (**C**). Dot plots show expression levels of represented markers within each cluster in the LD muscle. The percent expressed by the dot size and the shade of color represents average expression. (**D**). Bar graph of cell-type composition for each of the 14 samples. (**E**). Results of significantly enriched GO terms of the DEGs in 14 periods, and the column length represents the number of genes enriched into the GO terms. (**F**). Results of significant enriched KEGG pathways of the DEGs in 14 periods, and the dot size represents the number of genes enriched into the KEGG pathway. P-adjustments are represented by colors.

Single-cell suspensions from 14 periods of LD muscle were subjected to scRNA-seq using a single-tube protocol based on 10 × Genomics (Figure 2A) and gained 120,944 cells, with an average sequencing depth of 67,914 reads per cell, a sequencing saturation rate of 79.28%, and an average of 949 genes per cell. Cell clustering and UMAP visualization of single-cell transcriptomes were performed to identify different cell populations and cell heterogeneity after removal of low-mass cells and double cells. A total of 107,423 single cells from the different developmental time points were collected for the downstream analysis to continue (Supplemental Table S3).

UMAP identified a total of 25 major cell clusters based on their gene expression profiles (Figure S1B–N). Based on the specific marker genes for each cell population, we annotated the 13 major cell types, including skeletal muscle satellite cells (MuSCs), mitotic cells, myoblasts, muscle cells, fibro-adipogenic progenitors (FAPs), fibroblasts, endothelial cells, smooth muscle cells, tendon cells, and minor populations of immune cells, Schwann cells, neurons, and myeloid cells (Figure 2B and Figure S1B). The proportion of cells in different periods showed the time heterogeneity of LD muscle development (Figure 1B). We noticed a distinct change in the proportion of cell types during the maturation of LD muscle, with the number of cells of different cell types ranging from 379 to 23,192 (Figure 2D and Supplemental Table S3). The number of MuSCs and myoblasts gradually increased during embryonic development and decreased markedly after birth. The proportion of smooth muscle and endothelial cells rose after birth and in old age, accounting for more than 60%. FAPs persisted throughout growth, but the number and proportion of FAPs gradually declined over time. In late fetal and postnatal stages, myoblasts and fibroblasts declined, with few differentiated cells detected, likely due to incorporation of myogenic cells into multinucleated myofibers.

Although most of the marker genes were specifically or highly expressed in one cluster (for example, *PTPRC* in ICs), some were observed in a substantial proportion of cells in two or more clusters (for example, *COL1A1* in FAPs and TCs). This could be partly explained by the fact that some of the marker genes were labels for progenitors of multiple cell types (e.g., *PDGFRA*) and were not expressed in every cell in these clusters, which means that mature fibroblasts may be included in these clusters. Also, several cell clusters showed strong expression of multiple marker genes (Figure 2C). We discovered that the predominant regions of cell distribution in each stage were distinct, which fully substantiated that the evolution of the LD muscle is a dynamic process (Figure 2D).

For each developmental stage, we identified differentially expressed genes (DEGs) and performed Gene Ontology (GO) and KEGG pathway enrichment analyses, revealing that as the longissimus dorsi (LD) muscle matures, both GO terms and KEGG pathways exhibit distinct, stage-specific patterns (Figure 2E,F).

In Y11, DEGs are predominantly associated with “cell–substrate junction,” “focal adhesion,” and “regulation of apoptotic signaling pathway” in GO, while enriched KEGG pathways include “Chemical carcinogenesis-reactive oxygen species” and “Oxidative phosphorylation”, reflecting well-known processes of muscle aging such as altered energy metabolism and cell survival mechanisms. At the E30 stage (early embryos), DEGs were mainly enriched in GO terms like “Generation of precursor metabolites and energy”, “Transcription coregulator activity”, “RNA splicing” and “nuclear speck”, while KEGG pathways such as “Retrograde endocannabinoid signaling,” “Spinocerebellar ataxia,” and “Amyotrophic lateral sclerosis” were closely linked to early brain and limb development, in line with our previous observation that nerve cell populations peak around day 30 of gestation and indicate a rapid phase of neurogenesis and limb formation (Figure 2D). Finally, in the period spanning late embryonic (E120) to early postnatal (B90) stages, DEGs are enriched in GO categories including “cell–substrate junction,” “collagen-containing extracellular matrix,” and “extracellular structure organization,” with KEGG pathways such as “PI3K-Akt signaling,” “proteoglycans in cancer,” and “focal adhesion” underscore active cell proliferation, differentiation, and migration, which together demonstrate that LD muscle cells at these later stages are undergoing significant maturation and extracellular matrix remodeling.

### 3.3. Temporal Heterogeneity of MuSCs in Development

**Figure 3:**
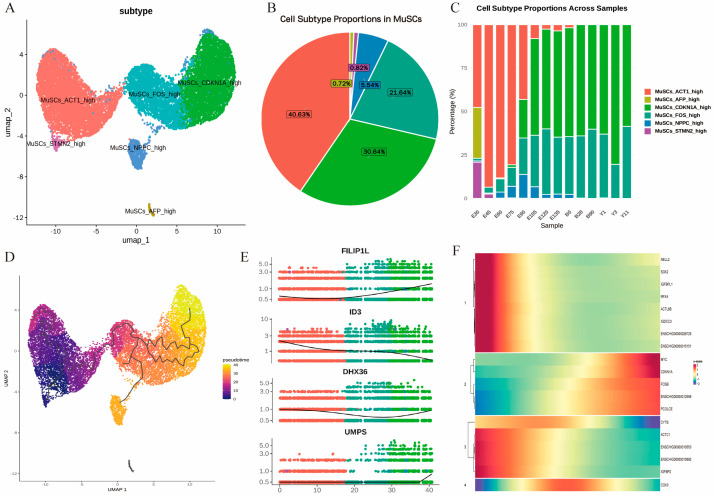
Subpopulation analysis and developmental trajectories of MuSCs across 14 developmental stages. (**A**). UMAP visualization of MuSCs subclusters, colored by identified subpopulations. (**B**). Proportion of each MuSCs subpopulation within the total MuSCs population. (**C**). Temporal dynamics of MuSCs subpopulation proportions across 14 developmental stages. (**D**). Pseudotime trajectory of MuSCs reconstructed using Monocle 3. (**E**). Expression dynamics of the differentially expressed genes along the inferred pseudotime. (**F**). Heatmap of differentially expressed genes identified in pseudotime analysis, clustered into four distinct modules, showing scaled expression (z-score) across pseudotime.

Skeletal muscle satellite cells serve as the primary myogenic progenitors in skeletal muscle, exhibiting distinct biological functions during the embryonic stage and postnatally. Accordingly, we initially prioritized the examination of heterogeneity and pseudotime developmental trajectories of skeletal muscle satellite cells across different periods. Through further dimensionality reduction and clustering of skeletal muscle subgroups, we annotated a total of six cell subpopulations: MuSCs_*ACT1*_high, MuSCs_*STMN2*_high, MuSCs_*FOS*_high, MuSCs_*NPPC*_high, MuSCs_*CDKN1A*_high, and MuSCs_*AFP*_high (Figure 3A and Figure S2A,B). Among these, MuSCs_*ACT1*_high, MuSCs_*CDKN1A*_high, and MuSCs_*FOS*_high accounted for the top three proportions in cell numbers across the 14 periods, suggesting that these three subpopulations may play predominant roles during these stages (Figure 3B).

The pseudotime analysis results were consistent with our findings. *ACT1*, as an early-emerging subpopulation, bifurcates into two branches: one differentiating into the *STMN2* subpopulation and the other into the *FOS* subpopulation. Within the *FOS* subpopulation, another differentiation branch emerges, with one part evolving into the *NPPC* subpopulation and the other into the *CDKN1A* subpopulation, which plays a role in aging skeletal muscle (Figure 3C). We subsequently analyzed the top 20 differentially expressed genes from the pseudotime analysis (Figure S2C), with particular emphasis on the notable temporal variations in *FILIP1L*, *ID3*, *DHX36*, and *UMPS* (Figure 3D). *FILIP1L*, serving as an aging-related marker gene, participates in biological processes such as suppressing excessive fibrosis and fat infiltration, thereby indirectly supporting skeletal muscle satellite cell-mediated muscle regeneration; however, its high expression can trap skeletal muscle satellite cells in a regenerative defect state. Both *ID3* and *DHX36* promote the proliferation of skeletal muscle satellite cells during skeletal muscle development, but in aged cells, downregulation of *ID3* may facilitate abnormal differentiation or lead to aging-related regenerative failure, thereby reducing skeletal muscle satellite cell reserves [20], whereas upregulation of *DHX36* in the later stages of development likely represents a compensatory increase in expression that fails to reverse functional decline, resulting in muscle homeostasis imbalance [21]. Although *UMPS* has not yet been reported to have a direct role in skeletal muscle satellite cell development, its high expression during terminal differentiation suggests potential indirect involvement in physiological processes such as aging skeletal muscle repair. Pseudotime trajectory analysis revealed dynamic gene expression changes in skeletal MuSCs during regeneration or development (Figure 3F). The heatmap clustered differentially expressed genes into four major clusters, each exhibiting a unique temporal expression pattern that reflects the continuous state transitions from quiescence to activation, proliferation, and differentiation. Genes in Cluster 1 (such as *NELL2*, *SOX2*, and *IGFBP1*) showed significant high expression in the early pseudotime phase, followed by a gradual decline, primarily enriched in stem cell population maintenance, regulation of stem cell quiescence, and signaling pathways regulating pluripotency of stem cells, indicating that these genes contribute to maintaining cellular quiescence and reserve states. Genes in Cluster 2 (such as *MYC*, *CDKN1A*, and *FOSB*) displayed peak expression in the mid-pseudotime phase, with notable enrichment in the cell cycle, p53 signaling pathway, MAPK signaling pathway [22,23], and immediate early response-related processes, suggesting that this stage primarily involves cell activation, proliferation initiation, and balanced cell cycle regulation. Genes in Cluster 3 and Cluster 4 (such as *ACTC1*, *CYTB*, *COX3*, and *IGFBP2*) underwent progressive upregulation in the late pseudotime phase, mainly enriched in muscle contraction, actin cytoskeleton organization, oxidative phosphorylation, and mitochondrial electron transport pathways, reflecting the transition from myogenic differentiation to the establishment of mature muscle fiber functions, along with metabolic reprogramming of energy during differentiation [24,25,26,27,28].

### 3.4. Temporal Heterogeneity of FAPs in Development

**Figure 4:**
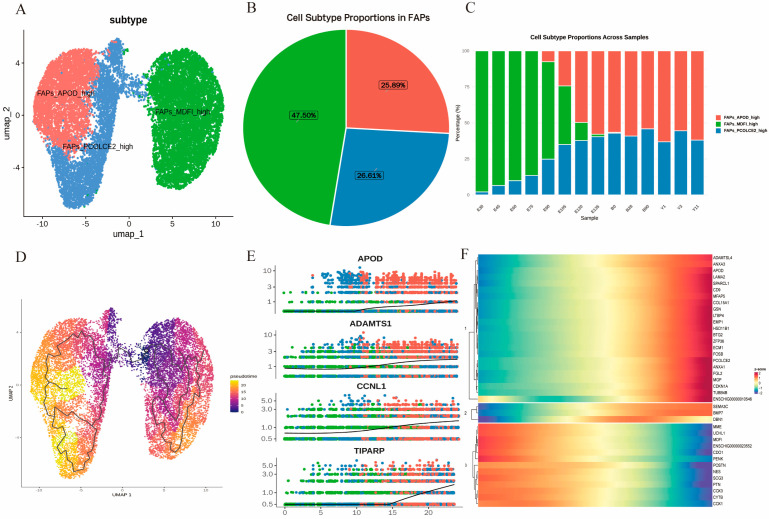
Subpopulation analysis and developmental trajectories of FAPs across 14 developmental stages. (**A**). UMAP visualization of FAPs subclusters, colored by identified subpopulations. (**B**). Proportion of each FAPs subpopulation within the total FAPs population. (**C**). Temporal dynamics of FAPs subpopulation proportions across 14 developmental stages. (**D**). Pseudotime trajectory of FAPs reconstructed using Monocle 3. (**E**). Expression dynamics of the differentially expressed genes along the inferred pseudotime. (**F**). Heatmap of differentially expressed genes identified in pseudotime analysis, clustered into four distinct modules, showing scaled expression (z-score) across pseudotime.

As mesenchymal-derived non-myogenic cells, FAPs possess the capacity to differentiate into adipogenic and fibrogenic precursors. During development, FAPs engage in bidirectional crosstalk with skeletal muscle satellite cells, participating in the regulation of multiple biological processes, including proliferation, differentiation, and senescence of satellite cells. Therefore, we focused on FAP subpopulations closely associated with skeletal muscle satellite cells and annotated three distinct subpopulations: FAPs_*MDFI*_high, FAPs_*PCOLCE2*_high, and FAPs_*APOD*_high (Figure 4A and Figure S3A,B). Among these, FAPs_*MDFI*_high accounted for the highest proportion at 47.50% (Figure 4B). Analysis of subpopulation dynamics across developmental stages revealed that the FAPs_*MDFI*_high subpopulation emerged specifically during the embryonic period and progressively decreased as embryonic development advanced. In contrast, FAPs_*PCOLCE2*_high began to increase from the E30 stage and maintained a relatively stable level postnatally. The FAPs_*APOD*_high subpopulation first appeared in the late embryonic stage (E90) and gradually increased throughout development, remaining at a consistent level after birth (Figure 4C).

Pseudotime trajectory analysis suggested potential differentiation paths consistent with subtype results. Starting from the *MDFI*-high subpopulation as the differentiation origin, two distinct branches emerged along the developmental trajectory: one progressing toward the *APOD*-high subpopulation and the other toward the *PCOLCE2*-high subpopulation, consistent with FAPs possessing the capacity to commit to fibrogenic and adipogenic lineages, respectively (Figure 4D). We further analyzed the top 20 differentially expressed genes along the pseudotime trajectory (Figure S3C), focusing particularly on *APOD*, *ADAMTS1*, *CCNL1*, and *TIPARP*. As previously reported, *APOD* upregulation is associated with adipogenic potential in FAPs. Its progressive increase along the trajectory is consistent with the accumulation of adipocyte-committed cells (Figure 4E). *ADAMTS1* promotes the activation of skeletal muscle satellite cells while simultaneously modulating the FAP microenvironment. Its increased expression suppresses excessive adipogenesis in FAPs, thereby maintaining adipose homeostasis—an observation that aligns closely with the relatively balanced proportions of FAPs_*PCOLCE2*_high and FAPs_*APOD*_high subpopulations in later developmental stages (Figure 4E). Intriguingly, *FILIP1L* also exhibited differential expression during FAP differentiation, with peak expression at the mid-stage followed by a decline in the late stage, suggesting its potential involvement in the fibrogenic differentiation of FAPs (Figure 4E). *TIPARP* has been reported to facilitate white and brown adipocyte differentiation by upregulating lipid accumulation-associated markers [29]; its elevated expression in the late differentiation phase further supports a commitment of FAPs toward an adipogenic fate (Figure 4E).

The pseudotime trajectory heatmap of FAPs revealed dynamic gene expression patterns across three distinct clusters, illustrating the progression from a quiescent/multipotent state to terminal differentiation (Figure 4F). Cluster 1 was characterized by late-stage upregulation of genes such as *ADAMTSL4*, *APOD*, *LAMA2*, *COL15A1*, and *PCOLCE2*, which are closely associated with processes related to extracellular matrix–receptor interaction, TGF-β signaling, PPAR signaling, extracellular matrix organization [30], and regulation of inflammatory responses [31,32,33,34]. These pathways predominantly drive fibrogenesis through enhanced ECM deposition and fibrosis, while partially supporting adipogenesis via lipid metabolism cues. This pattern may contribute to pathological intramuscular adipose tissue (IMAT) under chronic conditions. Cluster 2, defined by mid-stage peak expression of genes including *MME*, *MDFI*, *POSTN*, and *NES*, was closely associated with WNT and NOTCH signaling pathways [35,36,37,38], as well as processes related to negative regulation of cell differentiation and growth factor activity [39,40]. This cluster appears to function as a transitional “decision point,” wherein WNT-mediated inhibition of PPARγ restricts adipogenesis [41], whereas Notch/TGF-β crosstalk favors fibrogenesis in response to microenvironmental signals [42]. Cluster 3 was distinguished by early high expression of mitochondrial genes (*COX3*, *CYTB*, *COX1*), which are closely associated with oxidative phosphorylation, metabolic pathways, mitochondrial electron transport, and ATP synthesis [43,44]. This early metabolic reprogramming provides the energetic foundation for initial FAP activation, thereby facilitating subsequent adipogenic lipid synthesis or fibrogenic ECM remodeling; dysregulation of these processes during aging exacerbates fate imbalances toward fibrosis or fat infiltration. Collectively, these trajectories underscore the remarkable plasticity of FAP differentiation, wherein ECM remodeling and signaling pathways critically determine fibrogenic versus adipogenic outcomes in muscle homeostasis and pathology.

### 3.5. Convergent Transcriptional Regulatory Signatures Between MuSCs and FAPs Throughout Development

Transcription factors (TFs) play a critical role in regulating gene expression by specifically recognizing and binding to nucleotide sequences located upstream of their target genes.

**Figure 5:**
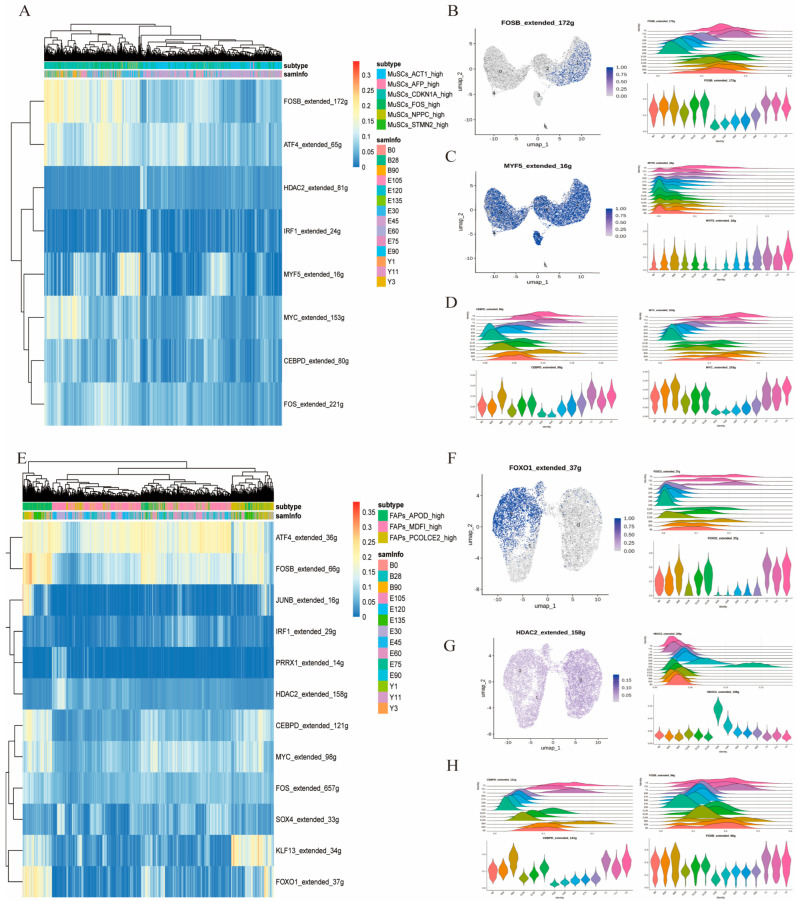
Transcription factor analysis of MuSCs and FAPs. (**A**) Heatmap showing regulon activity scores of the top transcription factors in MuSCs subpopulations identified by SCENIC, with rows representing transcription factors and columns representing individual cells ordered by subpopulation. (**B**) UMAP visualization and pseudotime trajectories of *FOSB*_extended_172g in MuSCs. (**C**) UMAP visualization and pseudotime trajectories of *MYF5*_extended_16g in MuSCs. (**D**) Pseudotime trajectories of *CEBPD*_extended_80g and *MYC*_extended_153g in MuSCs. (**E**) Heatmap showing regulon activity scores of the top transcription factors in FAPs subpopulations identified by SCENIC, with rows representing transcription factors and columns representing individual cells ordered by subpopulation. (**F**) UMAP visualization and pseudotime trajectories of *FOXO1*_extended_37g in FAPs. (**G**) UMAP visualization and pseudotime trajectories of *HDAC2*_extended_158g in FAPs. (**H**) Pseudotime trajectories of CEBPD_extended_121g and *MYC*_extended_66g in FAPs.

In the MuSCs population, eight transcription factors were identified (Figure 5A and Figure S2D), among which *FOSB*_extended_172g and *MYF5*_extended_16g were of particular interest due to their distinct expression dynamics and known biological relevance. *FOSB*_extended_172g exhibited elevated expression in the MuSCs_*CDKN1A*_high subpopulation. Although its expression was relatively low during early embryogenesis, it gradually increased throughout embryonic and postnatal development. Pseudotime analysis indicated that expression of this transcription factor was elevated in early cellular states during early embryogenesis (E30–E90) and subsequently showed peak expression in intermediate cellular states during later developmental stages (E105-Y11) (Figure 5B), consistent with a potential temporally dynamic role in MuSCs maturation. *MYF5*_extended_16g, a well-known myogenic regulator, was expressed across all five MuSCs subpopulations (Figure 5C). Given its essential role in muscle development, we specifically examined its temporal expression pattern. Pseudotime trajectory analysis showed that *MYF5*_extended_16g was elevated in early cellular states across all developmental stages. However, its regulon activity progressively declined during adulthood and aging, eventually reaching a silenced state. However, its expression progressively declined during adulthood and aging, eventually reaching low levels. Notably, its expression level gradually increased with development and peaked at the young adult stage (Y3) (Figure 5C), consistent with its involvement in early myogenic commitment and potential attenuation in mature and aging muscle.

In the FAPs population, a total of 12 transcription factors were identified (Figure 5E and Figure S3D). Among these, *ATF4*_extended_36g exhibited consistently high expression across all developmental stages and FAPs subpopulations (Figure S3E), suggesting a broad regulatory role. We further focused on two transcription factors with pronounced temporal specificity: *FOXO1*_extended_37g and *HDAC2*_extended_158g. *FOXO1*_extended_37g was specifically enriched in the FAPs_*APOD*_high subpopulation. Pseudotime trajectory analysis showed that expression of this transcription factor was relatively high during early embryogenesis (E30–E90) and subsequently increased over time. Despite this rise in expression, its regulon activity appeared to transition into a silenced state during later developmental stages (Figure 5F), suggesting potential stage-specific regulatory functions. In contrast, *HDAC2*_extended_158g displayed relatively low expression overall but demonstrated clear temporal activation patterns. During early embryonic development, it maintained a high expression level, which gradually decreased with developmental progression and stabilized around E135 (Figure 5G). Pseudotime analysis showed that *HDAC2*_extended_158g exhibited stage-specific expression patterns. Its expression was higher in mid-phase cells at E30 and E45, whereas in later stages, expression was mainly observed in early pseudotime states. These findings suggested that *HDAC2*_extended_158g may serve as a temporally restricted regulator of FAP differentiation and lineage commitment during skeletal muscle development. Additionally, several other transcription factors, including *KLF3*_extended_34g, *FOS*_extended_657g, *JUNB*_extended_16g, and *SOX4*_extended_33g, were found to play important roles in the differentiation of FAPs subpopulations (Figure S3F).

In particularly, several transcription factors identified in skeletal muscle satellite cells were also detected in FAPs, including *FOSB*, *ATF4*, *HDAC2*, *IRF1*, *MYC*, and *CEBPD* (Figure 5D,H, Figures S2E and S3E). *FOSB* exhibited a progressive increase in expression from early embryogenesis onward in both cell types, suggesting its potential role as a shared mediator of skeletal muscle growth, development, and adipogenesis in MuSCs and FAPs. *ATF4* was present in both populations, though its expression was consistently higher in FAPs than in satellite cells. *HDAC2* reached peak expression at the E30 stage in both cell types and subsequently declined as development proceeded. Furthermore, *IRF1*, *MYC*, and *CEBPD* displayed highly similar temporal expression patterns and comparable levels across the two populations. This convergence likely reflects the shared utilization of key signaling axes, such as the TGF-β pathway, in which *FOSB* serves as a common downstream effector. Such overlap strongly implies coordinated regulatory interplay between satellite cells and FAPs during development, underscoring the intricate crosstalk that governs their respective behaviors and fates.

### 3.6. Differences in Cell–Cell Communication During Development

**Figure 6:**
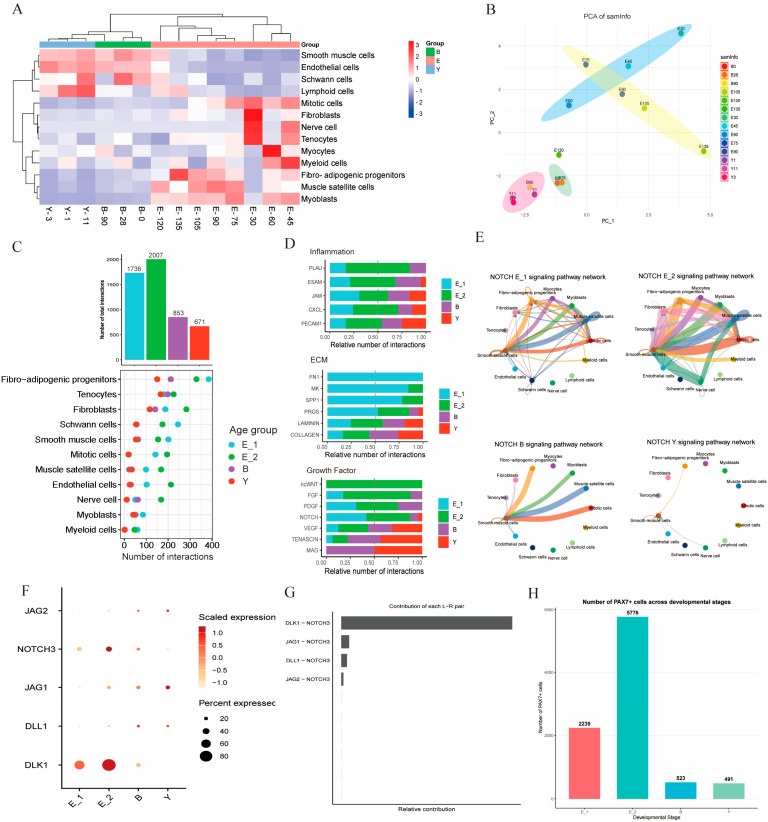
Intercellular communication analysis across four distinct age groups. (**A**). Analysis of correlations between cell types and individuals at 14 developmental stages. (**B**). PCA analysis of individuals across 14 developmental stages. (**C**). Bar plots and dot plots showing cell-type counts across the four age groups. (**D**). Four age groups of relative number of interactions in inflammation-, ECM-, and growth factor-related genes. (**E**). NOTCH signaling pathway interaction network of four age groups. (**F**). Scaled expression of ligand–receptor pair genes in the four age groups. (**G**). Contribution of each L-R pair in the E_2 age group. (**H**). Bar graph showing the number of PAX7^+^ cells across the four age groups.

To more clearly delineate the interactive relationships between skeletal muscle satellite cells and FAPs during development, we performed a detailed analysis of cell–cell interactions across 14 developmental periods. Initially, to better differentiate variations in cell interactions among distinct stages, we conducted correlation analysis (Figure 6A) and PCA clustering (Figure 6B) based on cell numbers and DEGs profiles at different stages, thereby reclassifying the 14 periods into four consolidated phases: E_1 (E30–E60), E_2 (E75–E135), B (B0–B28), and Y (B90–Y11).

We first compared differences in the number and strength of cell–cell communications across these four periods. The results of intercellular communication at four developmental stages showed that the total number of interactions between cells and the number of interactions between most cells reached a peak at E_2. The number of interactions between these cells gradually decreased with development (Figure 6C). The number of transmitted and received signals of each cell type in the four stages also showed that the interaction between multiple cells was significantly enhanced during E_2. Based on these results, we focused on the three interaction categories of inflammation, ECM, and growth factors to explore the developmental characteristics of the LD muscle during development (Figure 6D).

Inflammation during muscle injury is typically accompanied by the proliferation of MuSCs and FAPs, which support subsequent muscle repair and regeneration [42,45,46]. The statistical results of inflammatory pathways in the four periods showed that there was always a high interaction between inflammatory pathways during embryonic development (Figure 6D). In the inflammatory-related pathways, we observed that immune cells and myeloid cells increased the secretion of *CXCL*, and in embryonic myoblasts, the secretion of *JAM* also increased, both acting on a variety of cell types including muscle fibers. We observed the highest secretion of *CXCL* and *JAM* in the E_2 period, indicating that the muscle was at the peak of proliferation and differentiation (Figure S4C).

In recent years, NOTCH signaling has been demonstrated to play a critical role in early lineage commitment of FAPs, maintenance of quiescence in MuSCs, and related aspects of skeletal muscle homeostasis and regeneration [39,47,48]. Therefore, we focused on differences in cell–cell communication mediated by the NOTCH signaling pathway across four developmental stages. The results showed that during early embryonic development, the NOTCH pathway exhibited pronounced communication between FAPs and MuSCs. In contrast, this intercellular communication was abruptly lost after birth and during later developmental stages, suggesting that embryonic-stage interactions between these two cell types may play a critical role in regulating MuSC proliferation and differentiation (Figure 6E). Ligand–receptor pair analysis of the NOTCH pathway revealed that *DLK1* expression was significantly higher at the E_1 and E_2 stages than at the B and Y stages (Figure 6F), and the DLK1-NOTCH3 ligand–receptor pair contributed most strongly to overall NOTCH pathway activity (Figure 6G). Further analysis of DLK1-NOTCH3-mediated intercellular communication across the four developmental stages demonstrated that FAPs primarily interacted with MuSCs and smooth muscle cells (Figure S4B). Cell number analysis revealed that the number of *PAX7^+^* cells was significantly higher in the E_1 and E_2 stages compared to postnatal stages, indicating that most MuSCs remain in a quiescent state with limited differentiation capacity during the embryonic period (Figure 6H). These findings further corroborate our previous results and indicate that DLK1–NOTCH3 signaling plays a pivotal role in FAP-mediated regulation of MuSC proliferation and differentiation during embryonic development.

## 4. Discussion

### 4.1. The Heterogeneity of Goat Skeletal Muscle Cells in 14 Periods Characterizes the Development of Different Stages

The whole process of muscle development is well-known and occurs mainly before birth. It includes the production of primary muscle fibers and secondary muscle fibers in the early stage and the hypertrophy of muscle cells, adipose tissue, and fibrous tissue in the later stage. From the cellular level, the whole muscle development process can be found as muscle tissue is a highly heterogeneous tissue in nature. Myofibers are multinucleated cells; they are much longer and larger than monocytes. Despite their predominance in tissue, mature muscle fibers are a minority in single-cell studies because multinucleated cells are not easily isolated in single-cell isolation methods [9,10]. Although recent works have used scRNA-seq to outline skeletal muscle [4,5,9,10,49], in summary of recent work, muscle tissue has been profiled mainly by using mononuclear sequencing in postnatal or adult model mice [5,50,51,52]. In this study, we provided a comprehensive single-cell atlas of goat skeletal muscle development, spanning from embryonic day 30 to 11 years postnatal. The primary objectives of the experiment were to gain a comprehensive understanding of the cellular composition of muscle development in ruminants from the embryonic to postnatal stages and to elucidate the close functional relationships between satellite cells and other cell types throughout this process.

We annotated 13 cell types during muscle development. These include muscle components, the main smooth muscle cells, fibroblasts, and myoblasts, which play an important role in the movement and contraction of muscle and other basic functions. In addition, most of the cells detected here are progenitor cells such as MuSCs and FAPs. *PDGFRA* serves as a classic marker for fibro-adipogenic progenitors (FAPs) and their mesenchymal progenitors. However, mature fibroblasts, which often arise from FAP differentiation, display highly overlapping gene expression patterns with these progenitors (Figure 2C). This overlap likely contributes to the very limited representation of distinct mature fibroblasts within the clusters [49]. We identified several new isoforms in MuSCs based on their highly expressed genes (such as *ACT1*, *AFP*, *CDKN1A*, and *FOS*), which also provides strong support for the fiber specificity of muscle. These subtypes played an important role in muscle regeneration after injury. In addition, we observed some time specificity in the emergence of different cell types through the expression patterns of marker genes, cell trajectory analysis, and histological structure.

### 4.2. Temporal Specification of MuSCs and FAPs States Orchestrated Skeletal Muscle Development

MuSCs exhibit hierarchically regulated priming. The E30-restricted *STMN2*-high burst aligns with its role in microtubule disassembly, enabling rapid progenitor expansion [53]. Its neuronal-like expression pattern suggests conserved mechanisms in excitable-tissue progenitors, but aberrant persistence may underlie developmental myopathies. *ACT1* encodes skeletal muscle α-actin, a structural protein in muscle fibers that directly contributes myonuclei to form new muscle fibers [54]. Meanwhile, the MuSCs_*ACT1*_high subpopulation specifically emerges before B0 days and gradually decreases with advancing embryonic development time (Figure 3C), indicating that skeletal muscle satellite cells generate a substantial number of new muscle fibers during the embryonic period. *CDKN1A*, recognized as a reported marker of skeletal muscle aging [22,55,56], exhibits high expression that signifies cellular senescence and regeneration inhibition. Its appearance in late embryonic expression may reflect mutual regulation between *ACT1* and *CDKN1* in the late embryonic phase, thereby sustaining normal proliferation of skeletal muscle satellite cells during embryogenesis and preserving their quiescent state, whereas a substantial increase in adulthood implies that skeletal muscle satellite cells further progress into a senescent state, thereby suppressing their regeneration. As a subpopulation responsive to muscle injury or stimuli, the *FOS* subpopulation appears across all 14 time periods. Its continually increasing cell numbers from the embryonic stage through postnatally demonstrates that skeletal muscle satellite cells are driven by FOS throughout these 14 periods, prompting their exit from quiescence, entry into the G1 phase, and facilitation of activation along with early proliferation.

The temporal shift in FAP states reflected dynamic lineage priming. Within FAPs, *MDFI* primarily functions as a transcriptional repressor that inhibits the activity of myogenic factors such as *MyoD* [57]. The predominance of the *MDFI*-high subpopulation in early embryogenesis indicates that these cells represent a quiescent or early reserve state of FAPs lacking the capacity to differentiate into myofibers. *MDFI*-high fetal FAPs likely act as myogenic gatekeepers, transiently inhibiting differentiation to expand progenitor pools via *MyoD* suppression [58]. Their disappearance may “lock” postnatal FAPs into fibro-adipogenic fates, limiting innate regenerative capacity. *APOD*-high and *PCOLCE2*-high subsets emerge as effectors of niche maturation: *APOD*-mediated lipid shuttling may initiate adipogenesis, while *PCOLCE2*’s collagen processing could scaffold fatty infiltration. This adipogenic bias, while physiological during development, may become maladaptive in aging or metabolic disease [59]. The emergence of FAPs_*PCOLCE2*_high and FAPs_*APOD*_high subpopulations reflects two divergent differentiation trajectories. *PCOLCE2* is involved in collagen processing and extracellular matrix (ECM) remodeling and is commonly expressed in fibrotic or chondrogenic lineages, thereby marking an ECM-secretory and fibrosis-prone subpopulation. Its presence in embryonic FAPs suggests that a subset of these progenitors establishes the foundation for subsequent myofiber differentiation and spatial organization through fibrogenic potential. APOD (Apolipoprotein D), a lipoprotein implicated in lipid transport, antioxidative defense, and stress responses, is prominently expressed in adipose tissue and closely linked to adipocyte formation [60]. The appearance of the FAPs_*APOD*_high subpopulation in late embryogenesis indicates an alternative differentiation direction toward adipogenic precursors. Notably, adipogenic differentiation was not evident during early-to-mid embryonic stages (E30–E75) and stabilized at a moderate level postnatally. Collectively, these findings highlight the temporal and functional heterogeneity of FAPs and their coordinated roles in supporting skeletal muscle development.

### 4.3. MuSCs and FAPs Exhibit Similar Transcriptional Regulatory Patterns During Development

Single-cell analysis of the MuSCs population revealed eight transcription factors, with *FOSB*_extended_172g and *MYF5*_extended_16g distinguished by their dynamic expression profiles and pivotal roles in orchestrating MuSCs differentiation and myogenic commitment throughout development. *FOSB*_extended_172g showed elevated expression in the MuSCs_*CDKN1A*_high subpopulation. Its gradual increase in expression throughout embryonic and postnatal development, along with its high activity in early cellular states during early embryogenesis (E30–E90) and subsequent peak activation in intermediate cellular states during later developmental stages (E105–Y11), suggests a dynamic regulatory role in MuSCs maturation. This pattern hints that *FOSB* might be involved in the transition of MuSCs from a proliferative state to a more differentiated state as development progresses. Future research could explore how *FOSB*’s activity is regulated over time and its specific target genes in MuSCs, which would deepen our understanding of MuSCs development and regulation. *MYF5*_extended_16g, a well-known myogenic regulator [61] expressed across all five MuSCs subpopulations, has a crucial role in muscle development. The pseudotime trajectory analysis revealed its consistent activation in early cellular states across all developmental stages, with a progressive decline in regulon activity during adulthood and aging, eventually reaching a silenced state. However, its expression level peaked at the young adult stage (Y3), indicating its sustained involvement in early myogenic commitment followed by functional attenuation in mature and aging muscle. This is consistent with previous studies showing *MYF5*’s vital role in early muscle cell determination and differentiation [62]. The observed decline in *MYF5*’s activity in adult and aging muscle may reflect the reduced regenerative capacity of MuSCs with age. Further investigation into the mechanisms underlying *MYF5*’s functional attenuation could provide valuable insights for developing therapeutic strategies to enhance muscle regeneration in aging populations.

Our analysis of the FAPs population identified 12 transcription factors, with *FOXO1*_extended_37g and *HDAC2*_extended_158g exhibiting pronounced temporal specificity in regulating muscle cell differentiation and lineage commitment across developmental stages. Specifically, *FOXO1*_extended_37g and *HDAC2*_extended_158g showed significant temporal specificity among the identified transcription factors. *FOXO1* is known to play a crucial role in muscle cell differentiation by regulating genes like *MyoD* and *MyoG*, and it is also involved in metabolic regulation and stress responses of muscle cells [63]. This aligns with our finding that *FOXO1* was transcriptionally active during early embryogenesis (E30–E90), suggesting it may drive muscle cell differentiation via similar mechanisms. However, its regulon activity became silenced in later developmental stages, implying potential stage-specific regulatory functions of *FOXO1* in altering cellular stress resistance and modulating FAP functions. HDAC2, a histone deacetylase, affects gene expression by modulating histone acetylation levels and thus impacts cell proliferation, differentiation, and apoptosis [64]. It can interact with muscle-specific transcription factors to inhibit muscle cell differentiation [65]. Our results showed that *HDAC2* was highly expressed in early embryonic development, and its expression gradually declined thereafter, stabilizing around E135. Pseudotime trajectory analysis revealed strong stage-specific regulon activity of *HDAC2*. It was highly active in mid-phase cells at E30 and E45 but mainly restricted to early pseudotime states in later stages. This indicates that *HDAC2* may maintain cell proliferation in early stages by inhibiting differentiation and could participate in determining FAP lineage commitment in the mid-stages of skeletal muscle development, thus briefly but crucially regulating cell composition and function during muscle development.

Our analysis revealed that transcription factors such as *FOSB*, *ATF4*, *HDAC2*, *IRF1*, *MYC*, and *CEBPD* exhibited highly similar transcriptional regulatory patterns (Figure 5D,H, Figures S2E and S3E). These factors play critical roles in regulating key pathways including TGF-β, WNT, and NOTCH. Previous studies have demonstrated that MuSCs and FAPs share overlapping transcriptional regulatory profiles, with common regulatory networks governing MuSC regeneration and FAP differentiation fate [18,66,67], These findings further support our observations and, for the first time, show that MuSCs and FAPs in ruminants also display highly similar transcriptional regulatory patterns during development. This shared regulatory landscape collectively orchestrates the regenerative, differentiation, and aging trajectories of both MuSCs and FAPs in ruminants.

### 4.4. DLK1 Secreted by FAPs May Represent a Key Ligand Responsible for Inhibiting MuSCs Differentiation During Embryonic Stages

Cell communication occurs via the specific recognition and binding of ligands by cell surface receptors, resulting in the formation of receptor–ligand complexes to activate receptors [68]. Receptor activation leads to conformational changes and signal transduction. A cascade of intracellular signal amplification is initiated in target cells, which changes cell metabolic activity through activation, affects gene expression through gene expression of regulatory proteins, and changes cell shape or movement through cytoskeleton modification [69].

In the present study, considering the intimate correlation between the NOTCH signaling pathway and skeletal muscle development or aging [70], as well as its pronounced temporal specificity across the four distinct developmental stages, particular attention was devoted to scrutinizing the receptor–ligand interactions within this pathway. Delta-like canonical Notch ligand 1 (DLK1), a non-canonical NOTCH receptor, has been demonstrated to play a pivotal role in both adipogenesis and myogenesis [71]. Previous research has unveiled that *DLK1* can inhibit the differentiation of pre-adipocytes into mature adipocytes [72,73], a finding that is supported by our pathway strength analysis, which revealed that the expression of *DLK1* diminishes markedly in the later stages (Y). Additionally, it has been observed that *DLK1* is rapidly downregulated in the postnatal skeletal muscle of normal sheep and mice, a process that is crucial for maintaining normal myofiber levels and muscle quality [74,75]. Our results also demonstrated that the intercellular communication strength was high during the embryonic stage, but the interactions between DLK1 and NOTCH receptor–ligand pairs weakened postnatally.

Based on these observations, it is hypothesized that the high-strength interactions between DLK1 and NOTCH during early embryogenesis reflect a regulatory mechanism in which DLK1, secreted by fibro-adipogenic progenitors (FAPs), binds to the NOTCH3 receptor on the surface of skeletal muscle satellite cells (MuSCs). This interaction suppresses NOTCH signal activation, thereby maintaining MuSCs in a quiescent state, promoting their proliferation during embryonic development, and inhibiting premature differentiation. Through this mechanism, the MuSCs pool is expanded during embryogenesis, providing a sufficient cellular reservoir for subsequent myofiber formation. In contrast, the loss of DLK1-NOTCH3 interactions after birth releases the inhibitory constraint on myogenic differentiation, ensuring that MuSCs enter the differentiation and fusion stages required for postnatal muscle development.

## 5. Conclusions

This study constructed the first single-cell atlas of goat skeletal muscle spanning from embryonic day 30 to 11 years of age, identifying a total of 14 distinct cell types. Subpopulation characterization and pseudotime trajectory analyses of skeletal muscle satellite cells (MuSCs) and fibro-adipogenic progenitors (FAPs) revealed cell type-specific dynamic changes during development, as well as notable similarities in transcription factor-mediated regulatory programs between the two cell populations. These findings suggested that MuSCs and FAPs may participate in the regulation of cell fate through shared signaling pathways in ruminants. Intercellular communication analysis further identified that FAPs secrete DLK1, which acts on the NOTCH3 receptor expressed on MuSCs, thereby contributing to the suppression of MuSCs differentiation during early embryonic development. From a developmental perspective, this single-cell atlas provides a comprehensive characterization of the similarities and interactions between MuSCs and FAPs throughout skeletal muscle development in ruminants, offering a theoretical framework for elucidating the reciprocal regulatory mechanisms governing these two cell populations.

## Data Availability

The raw data of single-cell sequencing for skeletal muscle from Chengdu gray goats across 14 developmental stages have been deposited in the China National Center for Information Database under accession number [PRJCA049754]. The data will be released upon publication of the article.

## Supplementary Materials

The following supporting information can be downloaded at: https://www.mdpi.com/article/10.3390/cells15020206/s1, Figure S1: Sample collection and UMAP visualization and marker gene expression for cell clusters identified across 14 developmental stages in goat skeletal muscle.; Figure S2: Subpopulation analysis and associated transcription factors of MuSCs.; Figure S3: Subpopulation analysis and associated transcription factors of FAPs.; Figure S4: Sample collection and UMAP visualization and marker gene expression for cell clusters identified across 14 developmental stages in goat skeletal muscle.; Table S1: Metrics summary for each sample; Table S2: Filter conditions for each sample; Table S3: Gene marker for the celltype and the cell numbers at 14 timepionts in the skeletal muscle of goat.
