# Supplementary figures and images for: Single-Cell Sequencing Reveals the Crosstalk Between MuSCs and FAPs in Ruminant Skeletal Muscle Development
Source: Cells. 2026 Jan 22;15(2):206. doi: 10.3390/cells15020206 (PMC12839742; doi:10.3390/cells15020206)

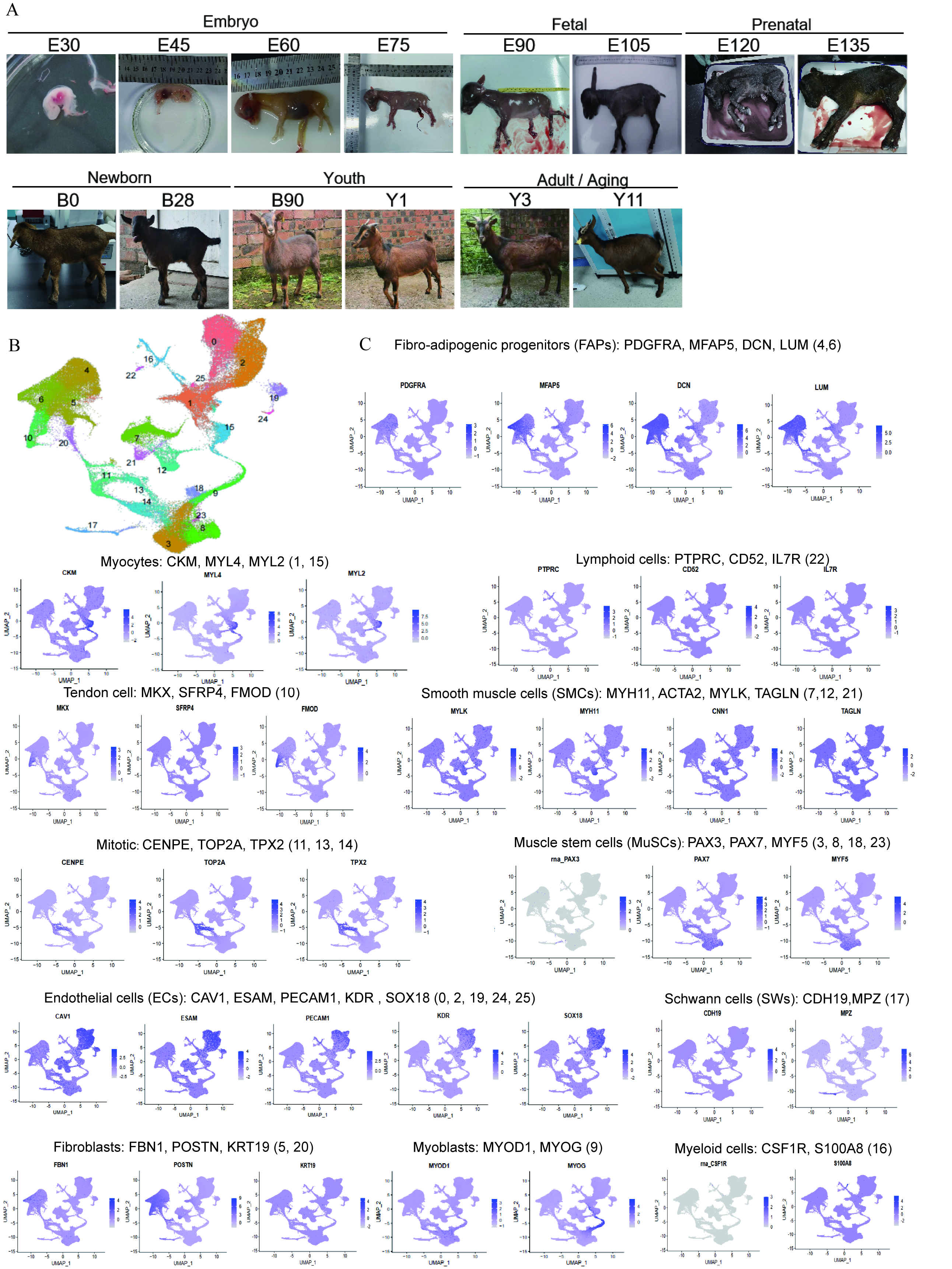

Supplement: Supplementary file 1 [file cells-15-00206-s001.zip › Figure S1.tif]

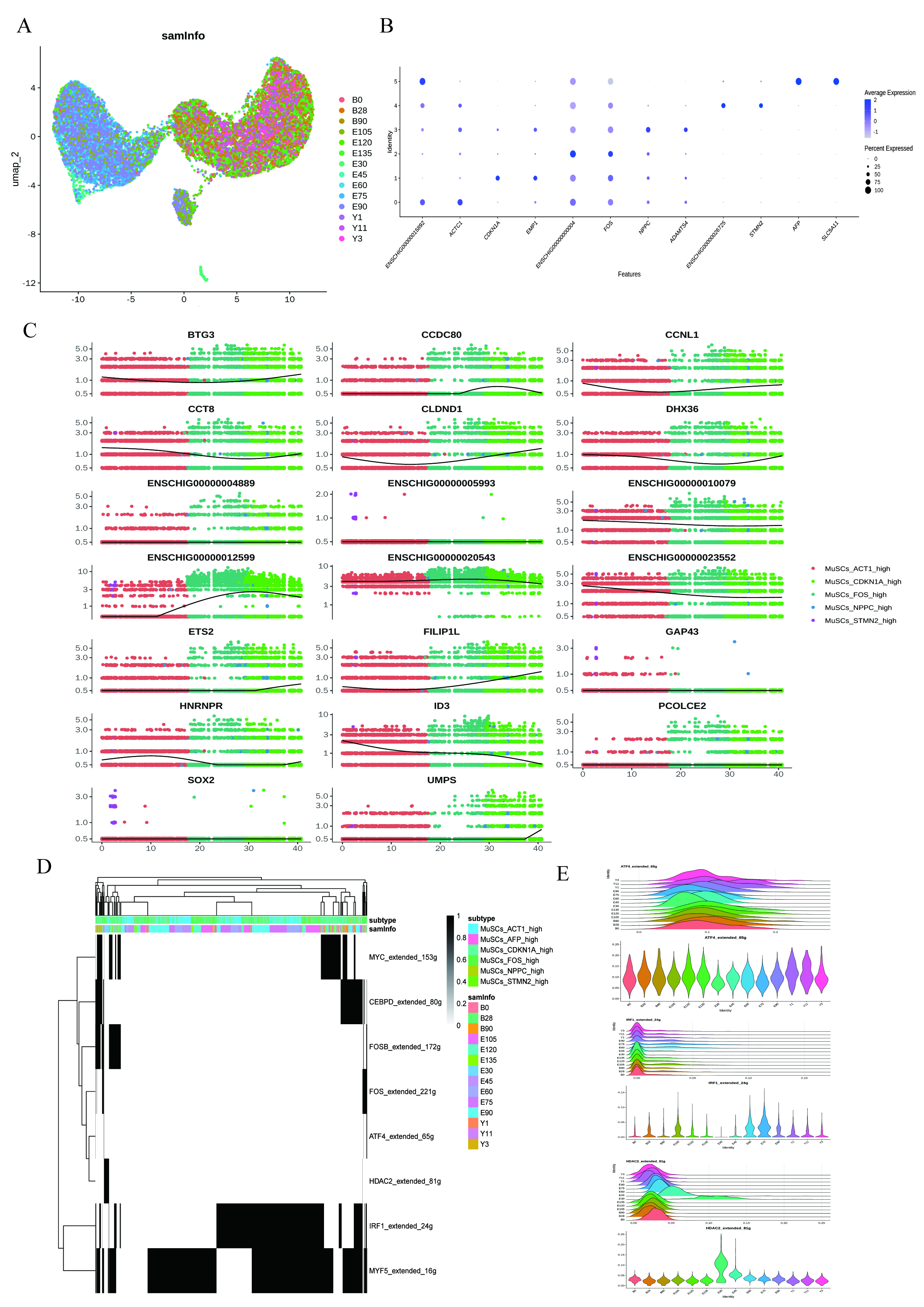

Supplement: Supplementary file 1 [file cells-15-00206-s001.zip › Figure S2.tif]

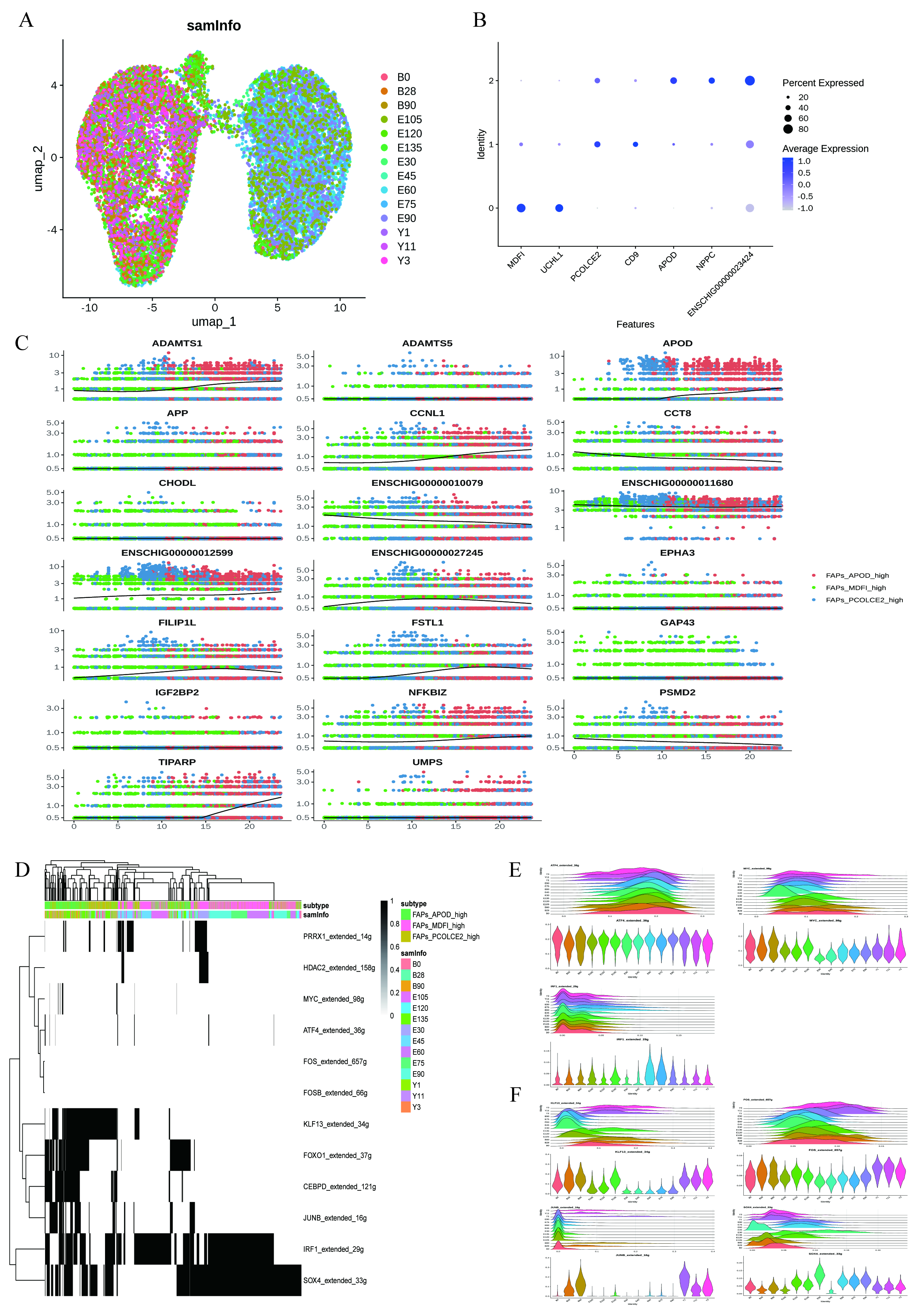

Supplement: Supplementary file 1 [file cells-15-00206-s001.zip › Figure S3.tif]

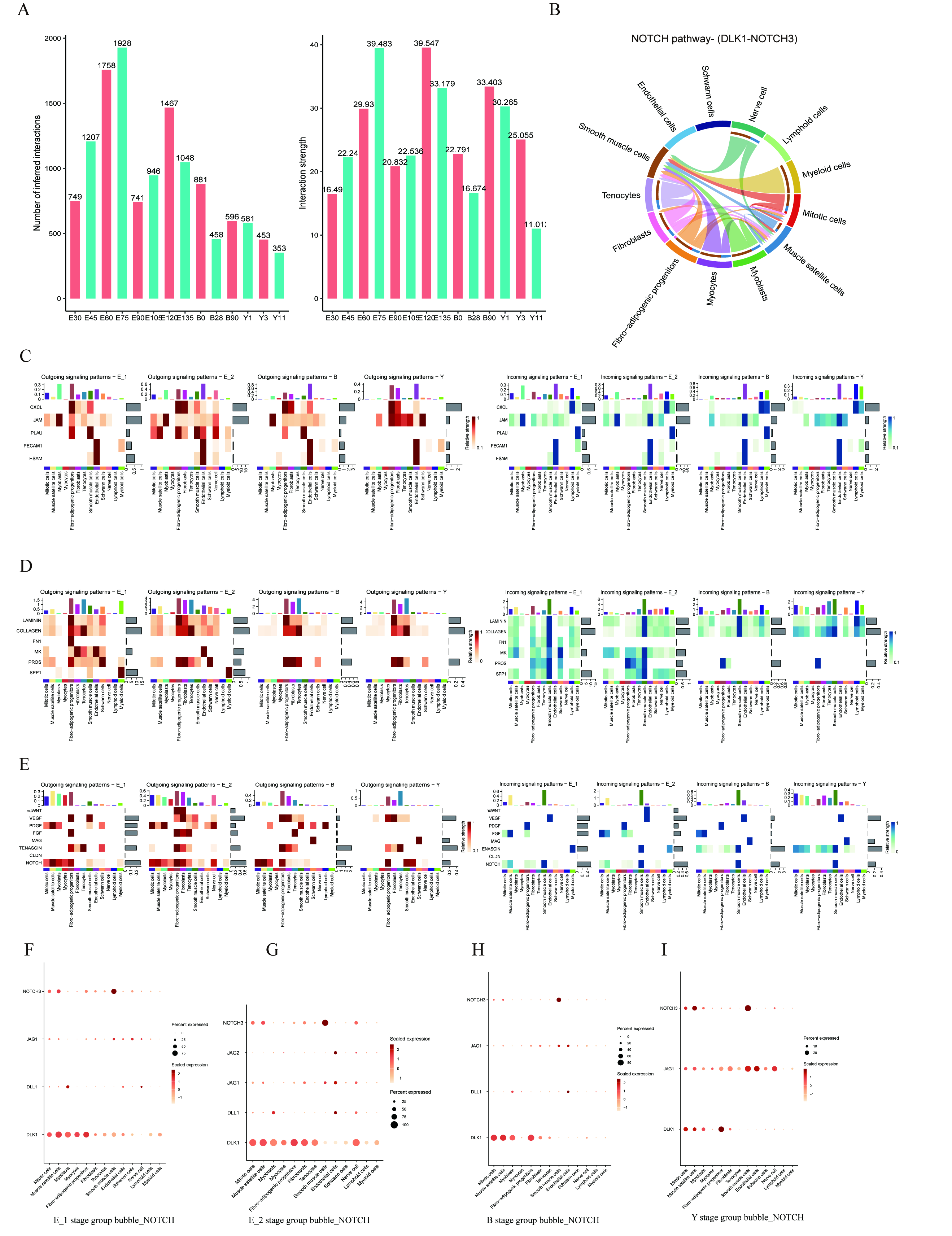

Supplement: Supplementary file 1 [file cells-15-00206-s001.zip › Figure S4.tif]
